# Revisiting EEG Biomarkers in Psychiatry: The Role of Sample Size in Reproducibility

**DOI:** 10.1192/j.eurpsy.2026.10251

**Published:** 2026-07-31

**Authors:** A. Ebadi, N. Le Guern, S. Kaderi, B. Rodríguez-Herreros, N. Chabane, S. Allouch, M. Hassan

**Affiliations:** 1 MINDIG, Cesson-Sévigné, France; 2 Electrical and Computer Engineering, Rafik Hariri University, Mechref, Lebanon; 3 Service des Troubles du Spectre de l’Autisme et apparentés, Département de psychiatrie, Lausanne University Hospital (CHUV), Lausanne, Switzerland; 4 School of Science and Engineering, Reykjavik University, Reykjavik, Iceland

## Abstract

**Introduction:**

Efforts to identify EEG biomarkers in psychiatry have been hampered by poor reproducibility and inconsistent findings, limiting their clinical translation. Underpowered studies, characterized by small sample sizes and unstable effect estimates, are thought to be a major contributor to this issue.

**Objectives:**

Here, we aim to systematically investigate how sample size affects the stability and replicability of group comparisons and brain–behavior associations in psychiatric EEG research.

**Methods:**

We analyzed a large cohort of ~2,900 participants, including individuals with neurodevelopmental or psychiatric conditions (ADHD, ASD, Anxiety, and Learning disorders) and healthy controls. Using iterative subsampling within each group, we conducted two analyses: (1) group comparisons with healthy controls via ANCOVA, controlling for age and sex, and (2) Spearman correlations between EEG features and clinical scores within each group. We examined trends in effect sizes and the significance of group differences across a wide range of sample sizes, from 10 participants to several hundred. The analyses covered 103 EEG features measured across 19 channels, with additional metrics averaged across all channels and 152 clinical scores.

**Results:**

At smaller sample sizes, significance detection was highly unstable, with results fluctuating across iterations (Fig. 1). We identified four distinct patterns in the evolution of significance across subsamples (Fig. 2a) and observed that overall stability improved with increasing sample size. Across disorders, the minimum sample size required for 95% reproducibility was substantially higher than the typical sample sizes used in EEG studies (Fig. 2b). Smaller samples exhibited high false-negative rates and inflated effect sizes, often overestimating and failing to detect true effects (Fig. 2c). These patterns were consistent across both group comparisons (ANCOVA) and within-group EEG–behavior correlations, highlighting the influence of sample size and clinical heterogeneity on the reliability of brain–behavior associations.

**Image 1:**
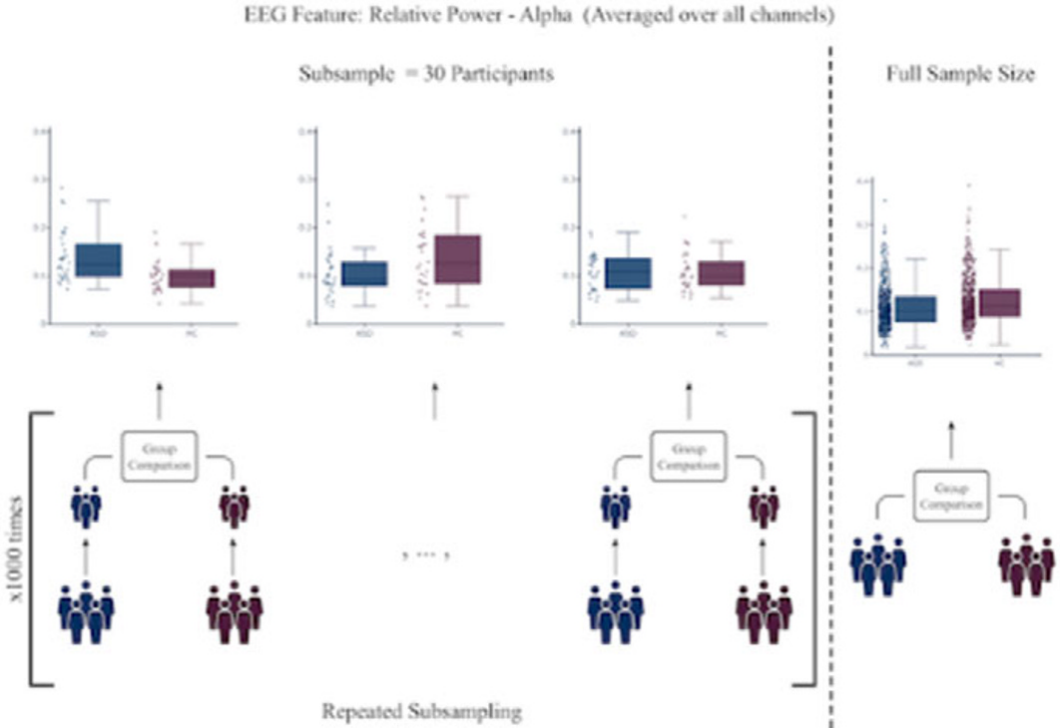
Image 1: Long description.

**Image 2:**
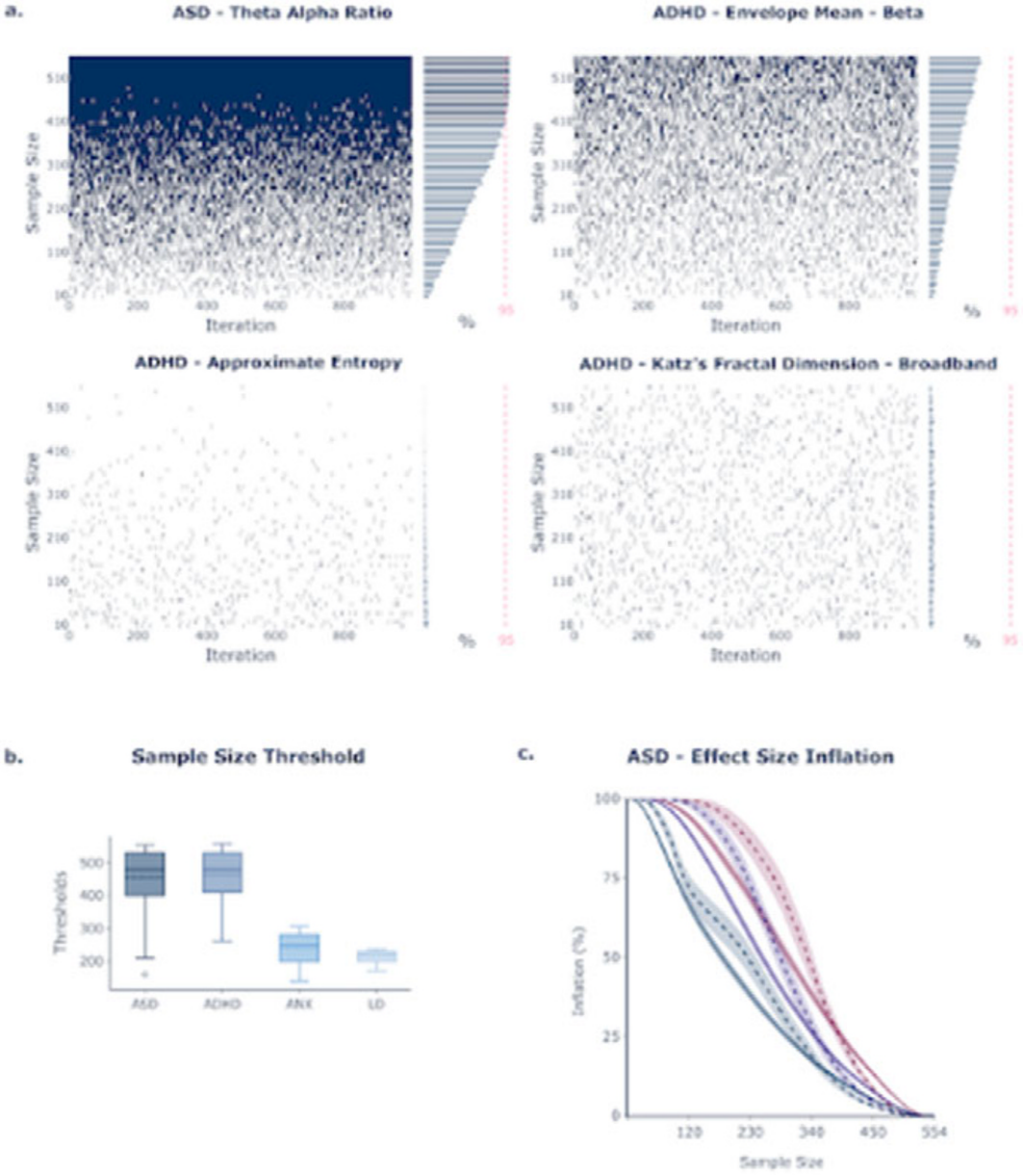
Image 2: Long description.

**Conclusions:**

Our findings demonstrate that typical EEG study sample sizes are insufficient to ensure reproducible results, leading to inflated or missed effects. Adequate scaling of cohorts is therefore essential for reliable biomarker discovery and clinical translation in psychiatry.

**Disclosure of Interest:**

None Declared

## Long descriptions

### Image 1: Long description

The diagram is divided into two main sections by a vertical dashed line.

Left Section: Repeated Subsampling

At the bottom, a bracket labeled x1000 times shows the process of Repeated Subsampling. It depicts two groups of human icons, blue for A S D and purple for H C. Arrows point from larger groups to smaller subgroups of 30 participants, which then lead to a Group Comparison box.

Above this process are three representative box plots. Each plot has a y-axis for relative power ranging from 0.0 to 0.4.

* The first plot shows A S D with a higher median and wider range than H C.

* The second plot shows H C with a higher median and wider range than A S D.

* The third plot shows both groups with similar medians and distributions.

Right Section: Full Sample Size

To the right of the dashed line, the full sample size is analyzed. At the bottom, the entire blue A S D group and purple H C group are fed into a Group Comparison.

Above this is a single box plot showing the aggregated data. The A S D and H C groups show heavily overlapping distributions with similar medians around 0.1 to 0.15, though the A S D group has a slightly higher density of individual data points at the lower end of the scale.

Navigate back to Image 1:.

### Image 2: Long description

Panel a contains four heatmaps arranged in a two by two grid. Each heatmap plots Sample Size from 10 to 510 on the y-axis against Iteration from 0 to 800 on the x-axis. To the right of each heatmap is a horizontal bar chart showing percentage.

* Top-left: A S D - Theta Alpha Ratio shows high density at larger sample sizes.

* Top-right: A D H D - Envelope Mean - Beta shows moderate density.

* Bottom-left: A D H D - Approximate Entropy shows very sparse data points.

* Bottom-right: A D H D - Katz's Fractal Dimension - Broadband shows very sparse data points.

Panel b is a box plot titled Sample Size Threshold. The y-axis is Thresholds from 100 to 500. The x-axis lists four groups: A S D, A D H D, A N X, and L D. A S D and A D H D have the highest thresholds, centered around 450 to 500. A N X is lower, around 250, and L D is the lowest, around 200.

Panel c is a line graph titled A S D - Effect Size Inflation. The y-axis is Inflation percentage from 0 to 100. The x-axis is Sample Size from 0 to 554. Multiple sigmoid curves descend from 100 percent toward 0 percent as sample size increases. The curves are color-coded in shades of blue, purple, and pink with shaded confidence intervals. The blue curves reach 0 percent inflation earliest, around a sample size of 450, while the pink curves reach 0 percent last, near 554.

Navigate back to Image 2:.

